# Sleep Problems and Quality of Life in Children with Epilepsy Without Neurodevelopmental Disorders

**DOI:** 10.3390/jcm13226892

**Published:** 2024-11-16

**Authors:** Kotaro Yazaki, Satoru Sakuma, Yuuto Shirokihara, Kayo Inutsuka, Takuji Imamura, Takenao Mihara, Naoko Tachibana, Kyoko Kondo, Wakaba Fukushima, Takashi Hamazaki

**Affiliations:** 1 Department of Pediatrics, Graduate School of Medicine, Osaka Metropolitan University, Osaka 545-8585, Japan; d20mb034@st.osaka-cu.ac.jp (K.Y.); hamazaki@omu.ac.jp (T.H.); 2 Department of Pediatrics, PL General Hospital, Osaka 584-8585, Japan; inutatu@gmail.com (Y.S.); kayo.19.25.intk@gmail.com (K.I.); tak.imamura@omu.ac.jp (T.I.); 3 Division of Sleep Medicine, Kansai Electric Power Medical Research Institute, Osaka 553-0003, Japan; takenao.mihara@gmail.com (T.M.); nanaosaka@aol.com (N.T.); 4 Research Support Platform, Graduate School of Medicine, Osaka Metropolitan University, Osaka 545-8585, Japan; kyou@omu.ac.jp (K.K.); wakaba@omu.ac.jp (W.F.); 5 Department of Public Health, Graduate School of Medicine, Osaka Metropolitan University, Osaka 545-8585, Japan

**Keywords:** sleep problems, children with epilepsy, quality of life, Kinder Lebensqualität Fragebogen (KINDL-R), Japanese Sleep Questionnaire for Elementary Schoolers (JSQ-ES)

## Abstract

**Background/Objectives**: Sleep problems affect the quality of life (QoL) and treatment prognosis of children with epilepsy (CWE). We analyzed sleep problems and QoL in CWE but without neurodevelopmental disorders, which affect sleep and QoL. We also examined discrepancies between child self-reports and parent proxy reports in QoL assessments. **Methods:** Thirty-two CWE in grades 2–6 (aged 7–12 years) in regular classes who attended Osaka Metropolitan University Hospital and PL General Hospital between January 2022 and August 2023 were compared with 21 children who had attended the hospitals for acute non-neurological disorders and had recovered (control group). Children with neurodevelopmental disorders, those unable to answer questionnaires, and those taking sleeping pills were excluded. Children in both groups completed the Kinder Lebensqualität Fragebogen (KINDL-R); their parents completed the KINDL-R and Japanese Sleep Questionnaire for Elementary Schoolers (JSQ-ES). **Results**: There were no significant differences in mean (±SD) JSQ-ES total scores between the epilepsy and control groups (71.6 ± 21.4 vs. 63.2 ± 15.2, respectively; *p* = 0.16). In the epilepsy group, there were no significant differences in total or subscale KINDL-R scores between children with (JSQ-ES ≥ 80) and without (JSQ-ES < 80) sleep problems. Correlation coefficients between child self-reports and parent proxy reports for KINDL-R total scores were 0.171 (*p* = 0.348) and 0.656 (*p* = 0.001) for the epilepsy and control groups, respectively. There was a significant difference between the total scores of children’s self-reports and parents’ proxy reports in the control (*p* = 0.008) group, but not in the epilepsy group (*p* = 0.837). **Conclusions**: Sleep problems may not have significant impacts on the QoL of CWE without neurodevelopmental disorders. Parents of CWE do not always correctly assess their children’s QoL, so it is important to ask both children and their parents to obtain a comprehensive picture of their QoL.

## 1. Introduction

Because epilepsy is often a life-long chronic disease, the goal of treatment is not only seizure control, but also improving a patient’s quality of life (QoL). The World Health Organization defines QoL as “the individuals perception of their position in life, in the context of culture and value systems in which they live, and in relation to their goals, expectations, standards and concerns” [1]. QoL has been conceptualized as a multidimensional construct encompassing multiple domains, and most of the QoL measurements include physical, social, psychological, and cognitive domains [2]. Assessment of children’s QoL has challenges associated with the unique characteristics of the pediatric population, including proxy reporting issues. Children’s QoL is usually assessed via child self-reports and/or parent proxy reports. However, consistent discrepancies have been reported between the two assessments [3,4,5], such as parents tend to rate their children’s QoL lower than the children themselves in the case of children with chronic health conditions. There is also a lack of evidence providing best-practice guidance on why, when, and how to use child self-reports and parent proxy reports in assessing QoL in child populations.

Sleep plays an important role in the normal development and daily life of children. Insufficient sleep is associated with a variety of physical, mental, and social problems, including obesity [6], depression [7], anxiety [8], impaired emotional regulation [9,10], poor cognitive abilities and academic performance [11,12], and poor relationships with friends and family [13], and can lead to lower QoL. Despite the importance of sleep to children’s health and QoL, Japanese children have been reported to be heavily sleep deprived and to experience sleep problems [14]. This may be truer for CWE, because a high percentage of these children are known to have sleep problems. Therefore, there is a pressing need to clarify the relationship between sleep problems and QoL in CWE. The etiology of sleep problems in CWE is multifactorial, including neurodevelopmental comorbidities, epileptic seizures, antiseizure medications (ASMs), and comorbid primary sleep disorders. Neurodevelopmental disorders such as autism spectrum disorder, attention-deficit/hyperactivity disorder (ADHD), and learning disability are common comorbidities in CWE [15,16,17] and related to both sleep problems and QoL [18,19,20]. Epileptic seizures disrupt sleep and alter subsequent sleep architecture. For example, in 24 patients with temporal lobe epilepsy, after nocturnal seizures, the N1 stage significantly increased from 11 ± 2% to 15 ± 2% (*p* = 0.002), REM sleep significantly decreased from 16 ± 1% to 6.8 ± 2% (*p* < 0.001), sleep efficiency significantly decreased from 91 ± 2% to 74 ± 4% (*p* < 0.001), and after daytime seizures, REM sleep significantly decreased from 18 ± 1% to 12 ± 2% (*p* = 0.003) [21]. The first day following status epilepticus was associated with a markedly abnormal sleep structure, consisting largely of the N1 stage with minimal slow wave sleep (SWS) or REM sleep, and over 4 days, SWS and REM returned to normal values [22]. On the contrary, in a survey of 104 epilepsy patients, 74 (71.2%) reported sleep deprivation as a precipitant of seizures [23], which is consistent with the results of a study showing that sleep deprivation activates epileptiform discharges independent of the activating effects of sleep [24]. A review of the effects of ASMs on sleep found that valproic acid increased the N1 stage and decreased SWS, clobazam decreased SWS, ethosuximide increased the N1 stage and decreased SWS, and phenobarbital prolonged sleep latency and dose-dependently decreased REM sleep, and when taken for a month, decreased the N3 stage, indicating that these classic ASMs are likely to have a negative effect on sleep. On the other hand, levetiracetam had heterogeneous results other than an increase in total sleep time (TST), lacosamide was harmless to sleep parameters, and perampanel decreased the N1 stage and increased SWS; these newer ASMs were less likely to adversely affect sleep [25]. CWE have a higher prevalence of primary sleep disorders, such as obstructive sleep apnea and insomnia [26,27,28]. Finally, it’s important to note that the relationship between epilepsy and sleep problems is bidirectional. Sleep problems can worsen seizure control, and poorly controlled epilepsy can further disrupt sleep, creating a vicious cycle [29].

Although ASMs are the first-line treatment for epilepsy, understanding and addressing sleep problems is therefore an important and treatable intervention target that could potentially improve children’s sleep, but also their seizures and QoL. A few studies have investigated the impact of sleep problems on QoL in CWE, all of which had a negative impact [30,31,32]. However, these studies include CWE and neurodevelopmental disorders. The impact of sleep problems on QoL is not uniform across all CWE and appears to vary with comorbidities. A recent study revealed that the impact of sleep problems on the QoL in CWE was, in fact, possibly mediated by neurodevelopmental characteristics [32]. Therefore, the aim of the present study was to investigate sleep problems and the QoL in CWE but without neurodevelopmental disorders. In addition, most of the previous studies evaluated the QoL in CWE using either child self-reports or parent proxy reports, and none examined the discrepancies between the two reports of the QoL in CWE without neurodevelopmental disorders. The secondary aim of the study is to investigate both child self-reports and parent proxy reports.

To assess the sleep problems, it is necessary to select a questionnaire that is appropriate for the child’s life. One of the most significant differences between the sleeping environments of Western and Japanese children is that in Japan it is common even for elementary school students to sleep with their parents on futons (mattresses laid out on the floor). Children may sleep alone when they fall asleep, but readily join their parents on their futons in the middle of the night. The Children’s Sleep Habits Questionnaire (CSHQ) [33] has been widely used to assess sleep habits in children in different countries, but it is based on Western culture. Liu et al. reported that co-sleeping affected the CSHQ [34]. In particular, the “sleep anxiety” items of these questionnaires are not designed for the Japanese sleeping environment. Therefore, it is appropriate to utilize the Japanese Sleep Questionnaire for Elementary Schoolers (JSQ-ES) [35,36,37], which was created by Mohri et al. to suit Japanese culture, rather than the CSHQ.

We tested the following hypotheses in this study: (1) sleep problems do not have a significant impact on the QoL of CWE without neurodevelopmental disorders, and (2) child self-report and parent proxy reports of the QoL of CWE without neurodevelopmental disorders do not agree.

## 2. Materials and Methods

### 2.1. Participants

The study was approved by the Ethics Committee of the Osaka Metropolitan University (protocol code 2022-090). This study included children in grades 2–6 (aged 7–12 years) in regular class who presented to the pediatric departments of Osaka Metropolitan University Hospital and PL General Hospital between January 2022 and August 2023. The former is a specialized medical institution with an epilepsy center and the latter is a regional core hospital with an outpatient pediatric neurology clinic. Children diagnosed with epilepsy were included as the epilepsy group; children who had attended the hospital with acute non-neurological diseases, such as respiratory tract infection and gastroenteritis, but had recovered and come for follow-up visits were included as the control group. Children with neurodevelopmental disorders, those who were unable to answer the questionnaire, and those taking sleeping pills were excluded from the study.

Children in both the epilepsy and control groups completed the Kinder Lebensqualität Fragebogen (KINDL-R; Children’s Quality of Life Questionnaire) [38,39], and their parents completed the KINDL-R and JSQ-ES [35,36,37]. Parents and children in the control group were asked to complete the questionnaires during a follow-up visit after the child had fully recovered from their acute illness.

**Figure 1:**
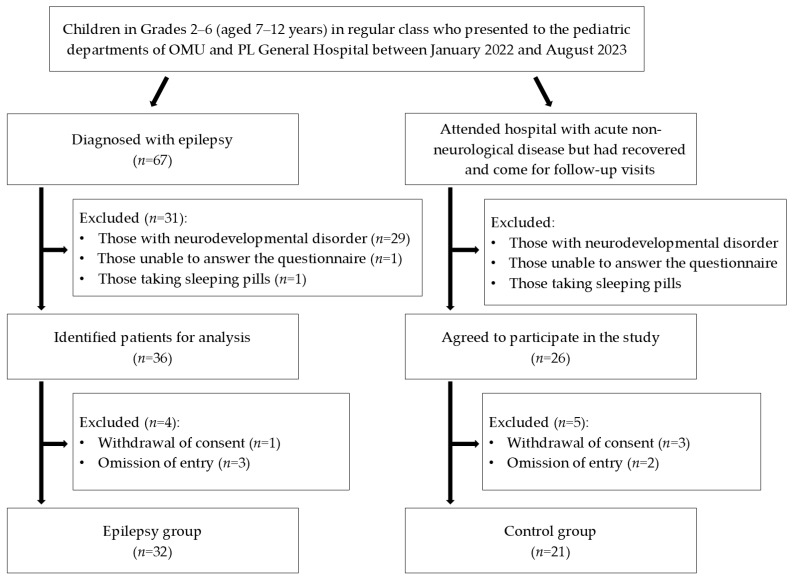
Patient flow diagram. Of the 67 children diagnosed with epilepsy, 35 in total were excluded, leaving 32 children in the epilepsy group. Of the 26 children eligible for inclusion in the control group, 5 were excluded, leaving 21 children in the control group. OMU, Osaka Metropolitan University Hospital.

A patient flow diagram is shown in Figure 1. Of the 67 children diagnosed with epilepsy, 31 met the exclusion criteria and were deemed ineligible for the study. Another 4 children were excluded after withdrawal of consent or a blank field; thus, the final epilepsy group consisted of 32 children. Of the 26 children eligible for inclusion in the control group who agreed to participate, 5 met the exclusion criteria; thus, the final control group consisted of 21 children.

### 2.2. Data Collection

Clinical data were collected for all patients and included sex, age, height, weight, and history of atopic dermatitis, allergic rhinitis, and asthma. For CWE, additional information was collected, including the duration of epilepsy, seizure-free period, seizure type, seizure frequency, and the number of ASMs. Data were obtained from the medical records of each hospital. We defined obesity as the percentage of overweight (POW) ≥20% (≥120% of the standard weight). In Japan, the POW measure is used in children more commonly than the body mass index for age percentile and is calculated based on measured weight and standard weight for height as follows: POW (%) = 100 × (measured weight − standard weight for height)/standard weight for height [40,41].

### 2.3. Characteristics

Patient characteristics are presented in Table 1. Male and female children were balanced in the epilepsy group (*n* = 16 each), but not in the control group (14 male, 7 female). The mean ages of the two groups did not differ significantly. Four children (12.5%) had obesity in the epilepsy group, compared with none in the control group (*p* = 0.092). Of the allergic diseases that could affect sleep and QoL [42,43], allergic rhinitis was more common in the control group, and there were no significant differences in atopic dermatitis and bronchial asthma between the epilepsy and control groups. The mean TST was significantly longer in the epilepsy than control group (*p* = 0.018). Of the 32 children in the epilepsy group, 5 (15.6%) had generalized seizures, 24 (75.0%) had focal seizures, and 3 (9.4%) had absence seizures. The frequency of seizures was daily to weekly in 2 children (6.3%), monthly in 6 (18.8%), and once a year or less in 24 (75.0%). With regard to ASMs, 11 children (34.4%) were taking multiple drugs, 20 children (62.5%) were taking one drug, and 1 child (3.1%) was not taking any ASMs. With regard to specific ASMs, 17 children were on levetiracetam, 7 children each were on sodium valproate and lacosamide, 5 were on perampanel, 3 each were on carbamazepine and lamotrigine, and 1 was on clobazam. Eight (25.0%) patients had sleep-related epilepsy, as defined by the International Classification of Sleep Disorders, Third Edition [44].

### 2.4. Measurements

#### 2.4.1. Japanese Sleep Questionnaire for Elementary Schoolers

The JSQ-ES is a sleep questionnaire for children adapted to Japanese culture created by Mohri et al. [35,36,37]. The questionnaire has been used in not only clinical but also research settings [45]. The target age range of the JSQ-ES corresponds to grades 1–6 of the Japanese school system (ages 6–12 years). On the front page of the JSQ-ES, parents answer questions about their child’s lifestyle habits (i.e., dinner time, bath time, co-sleeping status, wake-up time, and bedtime); on the back page, parents rate 38 items related to sleep problems (e.g., complaints about leg pain at bedtime, sleeps with his/her mouth open, grumpy in the morning, cries at night, oversleeps and is late for school, looks sleepy during the daytime, and falls asleep without any help) referring to the past month using a six-point Likert scale (ranging from “Not applicable at all” as 1 point to “Very applicable” as 6 points). Reversal items must be rescored before analysis. The scores for each item were added together, with higher scores on this scale indicating greater signs of sleep disorders or deleterious sleep habits. The questionnaire was validated using a large population-based sample of 4369 elementary school children (community group) and 100 children diagnosed with sleep disorders by professionals (clinical group), supporting its reliability across different populations [36]. The questionnaire showed high internal consistency, with Cronbach’s alpha values of 0.876 for the community group and 0.907 for the clinical group, indicating strong reliability across the questionnaire items. Exploratory factor analysis and confirmatory factor analysis suggested the following nine-factor structure covering a wide range of sleep-related issues: (1) “Restless legs syndrome”, consisting of six items suggesting restless legs syndrome such as pain, heat, and discomfort in the legs and increased activity at bedtime; (2) “Sleep-disordered breathing”, consisting of five items suggesting sleep disorder breathing such as sleeping with his/her mouth open and throwing his/her head back, snores loudly, stopping breathing frequently during sleep, and sleeping with snorts or gasps for air; (3) “Morning symptoms”, consisting of three items about morning symptoms related to decreased sleep quality and quantity such as difficulty waking up or morning irritability; (4) “Nighttime awakenings”, consisting of five items suggesting problems with maintaining sleep such as waking up at night due to crying or nightmares or being easily awakened by small noises due to shallow sleep; (5) “Insomnia”, consisting of three items often seen in children with insomnia such as reversal of day and night cycles, frequent tardiness, and absences from school; (6) “Excessive daytime sleepiness”, consisting of four items about symptoms of excessive daytime sleepiness resulting from decreased sleep quality and quantity such as sleepy or tired during the day and often snoozing at school; (7) “Daytime behaviors”, consisting of four items about daytime behavior problems resulting from decreased sleep quality and quantity such as restlessness, lack of concentration, irritability, and aggression toward others; (8) “Sleep habits”, consisting of two items about sleep habits such as going to bed by yourself and falling asleep alone; and (9) “Irregular/delayed sleep phase”, consisting of four items suggesting an irregular/delayed sleep phase such as going to bed more than one hour later or waking up more than one hour later on weekends and going to bed after 11:00 p.m.. To distinguish between clinical and community groups, the questionnaire has cut-off scores for the total score and the nine subscale scores mentioned above, allowing it to be used for comprehensive sleep evaluation and screening for individual sleep problems and sleep-related disorders. The cut-off score of 80 points for the JSQ-ES total score was determined with a sensitivity of 0.710 and specificity of 0.806 in the clinical group. This cut-off score corresponded to 20.1% of the community group. For comparison, the CSHQ cut-off score of 41 was determined with a sensitivity of 0.80 and specificity of 0.72 in a clinical group in Southeastern New England, corresponding to the top 23.2% of the community group [33]. Regarding the JSQ-ES subscales, all but “Irregular/delayed sleep phase” showed high sensitivity. However, it is not equivalent to a definitive diagnosis, and follow-up with diagnostic tests is necessary for an accurate assessment.

#### 2.4.2. Kinder Lebensqualität Fragebogen

The KINDL-R can assess the health-related QoL of children regardless of their health status in six different domains: “Physical wellbeing” (the child’s physical health status and functioning), “Emotional wellbeing” (the child’s emotional state and mental health), “Self-esteem” (the child’s sense of self-worth and confidence), “Family” (the quality of the child’s relationships and interactions within their family), “Social” (the child’s social relationships and interactions with peers), and “School” (the child’s performance and well-being in their school or educational environment) [38,39]. The KINDL-R has 24 items referring to the past week that are scored using a five-point Likert scale (ranging from “Never” as 5 points to “All the time” as 1 point). Reversal items must be rescored before analysis. The total score and each subscale were converted to a score ranging from 0 to 100, with higher scores reflecting a higher QoL. Examples of questions for each of the six domains include “I felt ill”, “I had fun and laughed a lot”, “I was proud of myself”, “I got on well with my parents”, “I played with friends”, and “Doing my schoolwork was easy”, respectively. The KINDL-R has demonstrated good psychometric properties, including satisfactory reliability and validity. It has been used in numerous studies and has been translated into multiple languages, making it a versatile tool for assessing QoL in children and adolescents across different cultures. We used the Japanese version of the Kid-KINDL-R [46]. The target age range of this questionnaire corresponds to grades 2–6 in the Japanese school system (ages 7–12 years). There are two versions of the questionnaire: one in which children rate their own QoL and another in which parents rate their child’s QoL. In the present study, both children and their parents were asked to complete the questionnaire. When children were completing the questionnaire, they were separated from their parents and completed the questionnaire in the presence of a medical professional.

### 2.5. Statistical Analysis

This was a cross-sectional, exploratory study, and no target sample size was set. Statistical analyses were performed using SPSS (version 23.0; IBM, Chicago, IL, USA). Categorical variables are presented as percentages and frequencies, whereas continuous variables are presented as the mean ± SD or as the median with interquartile range. Categorical variables were compared using the Chi-squared test and Fisher’s exact test. The distribution of continuous variables was examined using the Shapiro–Wilk test. Variables that were normally distributed were compared using Student’s *t*-test, whereas those that were not normally distributed were compared using the Mann–Whitney U test. Spearman’s rank correlation test was used to determine relationships between continuous variables. Wilcoxon’s signed-rank test was used for comparisons between two paired groups. All analyses were two-sided, and a *p*-value of <0.05 was considered statistically significant.

## 3. Results

### 3.1. Comparison of JSQ-ES Scores Between Epilepsy and Control Groups

The total JSQ-ES scores did not differ significantly between the two groups (*p* = 0.164; Table 2). Although the epilepsy group scored higher than the control group on the “Sleep-disordered breathing” (*p* = 0.044) and “Daytime behaviors” (*p* = 0.026) subscales, there were no significant differences between the two groups in the other subscales.

### 3.2. Impact of Sleep Problems on Kid-KINDL-R Scores in Children with Epilepsy

Table 3 and Supplemental Table S1 show a comparison of the Kid-KINDL-R scores between the groups above (indicating more likely to have sleep problems) and below the cut-off scores for the JSQ-ES total and each subscale score in the epilepsy group. There were no significant differences in total Kid-KINDL-R scores or in any of the subscale scores between those with a JSQ-ES total score of 80 or above and those with a score below 80 for evaluations either by the children themselves or their parents (Table 3). For any subscale of the JSQ-ES, there were no cases in which groups scoring above the cut-off score, i.e., more likely to have sleep problems, had significantly lower Kid-KINDL-R scores (Supplemental Table S1). Surprisingly, the child self-reported Kid-KINDL-R total score was significantly higher in the group whose “Morning symptoms” scores in the JSQ-ES were above the cut-off score (*p* = 0.040). In addition, the child self-reported “Family” score in the Kid-KINDL-R was significantly higher in groups whose “Sleep disordered breathing” and “Excessive daytime sleepiness” scores in the JSQ-ES were above the respective cut-off scores (*p* < 0.001 and *p* = 0.025, respectively), and the child self-reported “Self-esteem” score in the Kid-KINDL-R was significantly higher in the group whose “Daytime behaviors” scores in the JSQ-ES was above the cut-off score (*p* = 0.028).

### 3.3. Discrepancy Between Child Self-Report and Parent Proxy Reported Ratings on the Kid-KINDL-R

**Figure 2:**
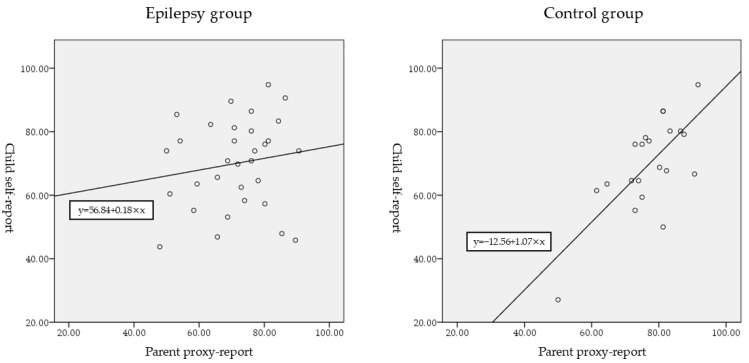
Scatterplots and regression lines of Kinder Lebensqualität fragebogen (Kid-KINDL-R) total scores for child self-reports and parent proxy-reports of quality of life in the epilepsy and control groups.

Self-reported and proxy-reported total Kid-KINDL-R scores were correlated in the control group (*ρ* = 0.656, *p* = 0.001), but not in the epilepsy group (*ρ* = 0.171, *p* = 0.348; Table 4; Figure 2). In terms of subscales, significant correlations were observed between self-reported and proxy-reported scores only for the “Social” subscale in the epilepsy group, and in the “Family” and “School” subscales in the control group.

Table 5 presents results of Wilcoxon’s signed-rank tests between child self- and parent proxy-reported ratings of Kid-KINDL-R scores in the epilepsy and control groups. There was a significant difference in total scores between the child self- and proxy-reported ratings in the control group, but not in the epilepsy group. With regard to subscales, significant differences were observed between self-reported and proxy-reported scores for the “School” subscale in the epilepsy group, and for the “Physical wellbeing” and “School” subscales in the control group.

## 4. Discussion

This was the first study to evaluate the impact of sleep problems on QoL and the discrepancy between child self-reports and parent proxy reports of a child’s QoL in CWE without neurodevelopmental disorders. Our results suggest that (1) sleep problems in CWE without neurodevelopmental disorders may be mitigated by good seizure control, use of ASMs with less impact on sleep, and longer TST with adequate sleep hygiene, (2) the negative impact of sleep problems on QoL in CWE may be partly due to the effects of comorbid neurodevelopmental disorders, and (3) child self-reports and parent proxy reports of a child’s QoL are also discrepant in CWE without neurodevelopmental disorders.

In the present study, 19.0% of children in the control group had a JSQ-ES total score higher than the cut-off value (80), indicating a higher level of sleep issues than typically observed in the general population, similar to the proportion reported by the authors of the JSQ-ES (20.1%) [36]. In the epilepsy group, the proportion was 18.8%, which was similar to the proportion in the control group. The mean total score on the JSQ-ES was slightly higher in the epilepsy than control group, but the difference was not statistically significant. Using the CSHQ and KINDL-R, Ekinci et al. compared 53 CWE and 28 children with mild medical problems (aged 7–18 years without intellectual disability) in Istanbul, Turkey [30]. CWE had a higher total CSHQ score than those in the control group. In the UK, Winsor et al. [32] asked parents of 36 CWE aged 4–16 years to complete the CSHQ, Social Communication Questionnaire (SCQ) [47], Conners 3 ADHD Index (Conners 3AI) [48], and the Quality of Life in Childhood Epilepsy Questionnaire (QOLCE-55) [49] and found a high prevalence (78.13%) of sleep problems in CWE. Stores et al. evaluated sleep disturbances in 79 CWE using a parent questionnaire and found a higher incidence of sleep problems (primarily poor sleep quality and anxiety about sleep) in CWE than in healthy controls [50]. The results of the present study differed from these previous studies. This is due, in part, to the exclusion of children with neurodevelopmental disorders. Other influencing factors include epileptic seizures, ASMs, and comorbid primary sleep disorders [21,22,23,24,25,26,27,28]. Our epilepsy group had a mean seizure-free period of 25.5 months, and the seizure frequency was once a year or less, monthly, and daily to weekly in 75.0%, 18.8%, and 6.3%, respectively, compared with 75.5%, 24.5%, and 0.0%, respectively, in Ekinci et al.’s study [30]. The good seizure control in our epilepsy group could be a factor in reducing the difference in JSQ-ES scores with the control group, but it was not a factor in explaining the difference in results from Ekinci et al.’s study [30]. Regarding ASMs, in Ekinci et al.’s study [30], 31 (58.5%) out of 53 patients were taking valproate and 12 (22.6%) were taking oxcarbazepine, whereas in our study, 50.0% were taking only newer ASMs which did not alter or significantly interfere with sleep architecture, such as levetiracetam, perampanel, and lacosamide [25]. Regarding primary sleep disorders, 12 (37.5%) and 7 (21.9%) patients exceeded the cut-off scores for the JSQ-ES subscales of “Sleep-disordered breathing” and “Insomnia”, respectively. These proportions do not differ significantly from previous reports [29]. Another notable result of this study was that the mean TST was significantly longer in the epilepsy than control group (*p* = 0.018; Table 1). In general, CWE are known to sleep less than healthy children [51]. Our results suggested that the shorter TST in children with epilepsy was partly due to mediation by neurodevelopmental disorders. It may also be influenced by our repeated instructions to keep regular hours, including going to bed early, during the treatment of CWE. In school-aged children, longer sleep duration was associated with better emotional regulation, better academic achievement, and better QoL [52]. Although the total KINDL-R scores assessed by the children did not differ between the control and epilepsy groups (69.9 ± 14.83 vs. 69.99 ± 13.98, respectively; *p* = 0.942), differences in TST need to be considered for interpretation. It should also be noted that the JSQ-ES includes questions directly related to TST, such as “Goes to bed after 23:00”. No previous studies have examined the extent to which TST affects total scores on the JSQ-ES. In the present study, a multiple regression analysis of the JSQ-ES total score was performed in the epilepsy group using TST and seizure-free period as explanatory variables, with the former being significant (*p* = 0.006) but the latter not. In addition, in the epilepsy group, gender, age, and obesity did not have a significant effect on the JSQ-ES total score. The epilepsy group scored significantly higher than the control group on the JSQ-ES subscales of “Sleep-disordered breathing” (*p* = 0.044) and “Daytime behaviors” (*p* = 0.026), but there were no significant differences between the two groups for the other subscales. The “Daytime behaviors” section of the JSQ-ES contains questions related to neurodevelopmental characteristics, such as “Restless in the daytime” and “Aggressive behavior towards others”, so it was surprising that significant differences were observed even after excluding children with neurodevelopmental disorders from the study. Children with neurodevelopmental disorders are more likely to have sleep-disordered breathing [53,54], but the results of the present study suggest that epilepsy itself may relate to sleep-disordered breathing and daytime behaviors. Future studies with a larger sample size are needed to clarify which characteristics of CWE are more closely related to these two subscales. Furthermore, although the JSQ-ES is a valid and reliable sleep questionnaire, it is only a parental assessment. Parental questionnaire ratings of their children’s sleep are less consistent with objective sleep measurements, such as actigraphy or polysomnography, in both healthy children and children with epilepsy [55,56,57,58,59]. For a more accurate understanding of sleep problems, these measurements should be considered in future studies.

Ekinci et al. reported that CWE with a CSHQ score >56, which indicates moderate to severe sleep problems, had lower scores on the KINDL-R [30]. In eastern India, Joseph et al. [31] asked the parents of 50 CWE aged 4–18 years to complete the 16-item Quality of Life in Childhood Epilepsy Questionnaire (QOLCE-16) [60] and CSHQ and found a significant negative correlation between QoL and sleep disturbances in CWE (r = −0.65, *p* ≤ 0.001). Winsor et al. also found that sleep problems had a clear negative impact on QOLCE-55 scores in CWE [32]. Incidentally, the respondents to both the QOLCE-16 and QOLCE-55 were the parents, not the children themselves. Unlike these previous studies, none of the various types of sleep problems had a significant negative impact on child self- and parent proxy-reported Kid-KINDL-R scores in CWE in the present study. This is probably because our study excluded children with neurodevelopmental disorders and may also be due, in part, to differences in age range, the questionnaires used, and cultures. In fact, Winsor et al. suggested that the negative impact of sleep problems on QoL in CWE may actually be mediated by comorbid neurodevelopmental disorders [32]. In addition, age and gender, which affect both the JSQ-ES and KINDL-R scores [36,46], seizure types and frequencies, ASMs taken, and comorbidities such as obesity and allergic diseases were not necessarily matched between the groups, which may also contribute to the result. Surprisingly, the child self-reported Kid-KINDL-R total score was significantly higher in the group whose “Morning symptoms” scores in the JSQ-ES were above the cut-off score, i.e., more likely to have sleep problems (*p* = 0.040). However, it is unlikely that morning symptoms related to decreased sleep quality and quantity such as difficulty waking up or morning irritability may actually have a positive effect on children’s QoL. In this study, as many as 18 (56.3%) CWE were above the cut-off score in the “Morning symptoms” subscale (Supplemental Table S1). It is possible that parents of CWE overestimated their children’s morning symptoms, resulting in children with only mild symptoms being included in the group with sleep problems. To obtain more robust evidence on the impact of sleep problems on QoL in CWE, we need a more objective and accurate diagnosis of sleep problems than a questionnaire assessment and a comparison of QoL in a larger sample size of CWE with similar backgrounds.

There are three general agreements regarding discrepancies in the QoL between parents and children [4]. First, parent proxy reports of QoL tend to be higher than child self-reports for healthy children. Conversely, for children with chronic health conditions, parents tend to rate their child’s QoL lower than the children rate themselves. Second, parents are usually better at reporting on their children’s observable behaviors than on their children’s internal states and emotions. Third, parent proxy reports of their child’s QoL are associated with their own QoL ratings. In the epilepsy group, Wilcoxon’s signed-rank test showed no difference between the child self-report and parent proxy reports in the total Kid-KINDL-R score. However, this only indicated that mean scores were not sufficiently different to be significant and did not indicate the relationship between scores across the cohort. In fact, the scores of the two groups were not correlated in the epilepsy group. The only subscales with significant correlations and no significant differences (considered as no parent–child discrepancy) were the “Social” subscale in the epilepsy group and the “Family” subscale in the control group. Parents in the control group rated their children’s Kid-KINDL-R total score higher, which is consistent with the consensus noted above. However, there was no significant difference between child self- and parent proxy-rated Kid-KINDL-R total scores in the epilepsy group. A possible reason is that 75.0% of the children had seizures less than once a year. In fact, Baca et al. reported that parent proxy reports of QoL in CWE were significantly associated with the 5-year seizure-free period, but child self-reports were not [61]. “Social” subscale consists of the following four items, which are largely based on the subjective perceptions of the children. (1) I played with friends. (2) Other kids liked me. (3) I got along well with my friends. (4) I felt different from other children. Thus, it was surprising that there was no apparent discrepancy in the “Social” subscale in the epilepsy group, although not a strong correlation (*ρ* = 0.405, *p* = 0.021). On the other hand, the “Family” subscale consists of the following four items that parents are likely to see and experience together. (1) I got on well with my parents. (2) I felt fine at home. (3) We quarrelled at home. (4) My parents stopped me from doing certain things. In the control group, the correlation was the strongest among the subscales (*ρ* = 0.624, *p* = 0.002), and the absolute value was not significantly different (*p* = 0.627). However, no correlation was found in the epilepsy group (*ρ* = 0.218, *p* = 0.231), and the difference in the absolute values, although not significant, was also noticeable (*p* = 0.053). Although child self-reports and parent proxy reports tend to agree on observable QoL domains even in CWE [62], the results in the present study were different. Further studies with a larger number of CWE are needed to determine in which domains the discrepancy is more pronounced in a population with any characteristics (types of seizure, age, gender, presence of neurodevelopmental disorders, presence of sleep disorders, etc.). Consistent with previous reports, our findings in this study suggest that parent proxy reports do not always accurately capture children’s QoL. There is a consensus that self-reporting should be used whenever possible when assessing the QoL of children as they reflect the child’s direct experience and perceptions [63]. However, parent proxy reports are also useful in many situations. It is often the parent’s perception of the child’s QoL that influences clinical decision-making and home care [64]. Proxy assessment is necessary for very young children or older children with health conditions or intellectual/cognitive impairment [65]. Children with neurodevelopmental disorders have problems with self-awareness and interpersonal understanding, making it difficult to assess their QoL on a scale that uses their own subjectivity [66]. Thus, in CWE, child self-reports and parent proxy reports do not always agree and have their own strengths and limitations, so we believe that asking both the parent and the child will lead to a comprehensive understanding of the child’s QoL and better treatment.

The strengths of this study include the exclusion of children with neurodevelopmental disorders, comparison to a control group, and assessment of QoL by the children themselves. However, this study has several limitations. First, the sample size was small compared with previous similar studies because of the stringent inclusion criteria. Sex, age, hospital attended, and the percentage of children with obesity and allergic diseases were not perfectly matched between the two groups. The mental state and QoL of the parents were not assessed. As mentioned above, we instruct CWE to keep regular hours for seizure control, and many CWE are taking ASMs, which may affect their sleep and QoL. Sleep assessment was based on parental responses to a questionnaire, and no objective sleep assessment was conducted. Finally, because this study was conducted during the COVID-19 pandemic, activities were restricted in many settings of daily life, which may have affected the QoL of the children in both groups.

## 5. Conclusions

Sleep problems may not have significant impacts on the QoL of CWE without neurodevelopmental disorders. Parents of CWE do not always correctly assess their children’s QoL, so it is important to ask both the children and their parents to obtain a comprehensive picture of their QoL. Effective treatment of epilepsy requires good sleep; so, when treating CWE, it is important to address sleep problems while paying attention to neurodevelopmental comorbidities.

## Figures and Tables

**Table 1:** Patient characteristics.

	Epilepsy Group (*n* = 32)	Control Group (*n* = 21)	*p*
Hospital			
OMU (*n*)	20	7	
PL General Hospital (*n*)	12	14	
Sex			
Male (*n*)	16	14	0.231
Female (*n*)	16	7	
Age (years)			
Mean ± SD	10.75 ± 1.70	10.48 ± 1.54	
Median [IQR]	11 [9,10,11,12]	11 [9,10,11,12]	
Height (cm)	137.8 ± 11.1	138.5 ± 11.7	
Weight (kg)	34.6 ± 10.4	32.6 ± 8.0	
Obesity	4 (13)	0 (0)	0.143
Sleep-related epilepsy (ICSD-3)			
Yes	8 (25)		
No	24 (75)		
Duration of epilepsy (months)	50.4 ± 29.7		
Seizure-free period (months)	25.5 ± 26.2		
Atopic dermatitis	1 (3)	2 (10)	0.152
Allergic rhinitis	2 (6)	5 (24)	0.031
Asthma	2 (6)	1 (5)	0.396
Total sleep time (h)	9.25 ± 0.76	8.79 ± 0.51	0.018

Unless indicated otherwise, data are given as the mean ± SD or *n* (%). OMU, Osaka Metropolitan University Hospital; ICSD-3, International Classification of Sleep Disorders, Third Edition.

**Table 2:** Comparison of JSQ-ES scores between epilepsy and control groups.

	Epilepsy Group (*n* = 32)	Control Group (*n* = 21)	*p*
JSQ-ES total score	71.60 ± 21.40	63.19 ± 15.16	0.164
JSQ-ES subscales			
Restless legs syndrome	8.22 ± 5.15	7.00 ± 2.14	0.492
Sleep-disordered breathing	9.81 ± 4.29	7.57 ± 2.99	0.044
Morning symptoms	8.94 ± 3.96	9.71 ± 3.80	0.494
Nighttime awakenings	7.50 ± 3.57	6.38 ± 2.27	0.138
Insomnia	3.63 ± 1.36	3.29 ± 0.72	0.672
Excessive daytime sleepiness	7.28 ± 3.71	5.90 ± 2.12	0.145
Daytime behaviors	11.03 ± 4.59	8.05 ± 3.49	0.026
Sleep habits	5.28 ± 3.76	5.05 ± 3.25	0.852
Irregular/delayed sleep phase	9.81 ± 4.92	10.24 ± 3.86	0.523

Unless indicated otherwise, data are given as the mean ± SD. JSQ-ES, Japanese Sleep Questionnaire for Elementary Schoolers.

**Table 3:** Comparison of Kid-KINDL-R scores in the epilepsy group between those with (JSQ-ES ≥ 80) and without (JSQ-ES <80) sleep disorder.

	JSQ-ES < 80 (*n* = 26)	JSQ-ES ≥ 80 (*n* = 6)	*p*
Child self-report			
Kid-KINDL-R total score	69.19 ± 14.75	73.44 ± 10.31	0.424
Kid-KINDL-R subscales			
Physical wellbeing	74.52 ± 19.68	73.96 ± 22.85	0.957
Emotional wellbeing	79.33 ± 17.39	82.29 ± 18.29	0.728
Self-esteem	55.53 ± 25.02	63.54 ± 13.36	0.293
Family	69.95 ± 16.87	83.33 ± 14.61	0.084
Social	74.52 ± 22.43	66.67 ± 25.52	0.510
School	61.30 ± 23.45	70.83 ± 13.50	0.207
Parent proxy report			
Kid-KINDL-R total score	72.12 ± 12.07	67.36 ± 10.51	0.359
Kid-KINDL-R subscales			
Physical wellbeing	76.92 ± 15.79	79.17 ± 21.16	0.815
Emotional wellbeing	80.53 ± 13.27	72.92 ± 12.29	0.215
Self-esteem	60.10 ± 18.46	58.33 ± 12.29	0.781
Family	67.07 ± 14.64	61.46 ± 7.31	0.194
Social	74.28 ± 21.89	64.58 ± 10.21	0.123
School	73.80 ± 15.00	67.71 ± 20.70	0.520

Unless indicated otherwise, data are given as the mean ± SD. JSQ-ES, Japanese Sleep Questionnaire for Elementary Schoolers; KINDL-R, Kinder Lebensqualität Fragebogen.

**Table 4:** Spearman’s rank correlation coefficients for total and subscale Kid-KINDL-R scores for the epilepsy and control groups.

	Epilepsy Group (*n* = 32)		Control Group (*n* = 21)	
	*ρ*	*p*	*ρ*	*p*
Kid-KINDL-R total score	0.171	0.348	0.656	0.001
Kid-KINDL-R subscales				
Physical wellbeing	0.194	0.287	0.416	0.061
Emotional wellbeing	0.062	0.737	0.327	0.148
Self-esteem	0.057	0.756	0.289	0.204
Family	0.218	0.231	0.624	0.002
Social	0.405	0.021	0.270	0.236
School	0.332	0.064	0.586	0.005

KINDL-R, Kinder Lebensqualität Fragebogen.

**Table 5:** Wilcoxon’s signed-rank test between child self- and parent proxy-reported ratings of Kid-KINDL-R scores in the epilepsy and control groups.

	Child Self-Reports		Parent Proxy-Reports		*p*
	Mean ± SD	Median [IQR]	Mean ± SD	Median [IQR]	
Epilepsy group (n = 32)					
Kid-KINDL-R total score	69.99 ± 13.98	72.36 [58.85–80.99]	71.22 ± 11.79	72.40 [64.06–80.21]	0.837
Kid-KINDL-R subscales					
Physical wellbeing	74.41 ± 19.91	75.00 [56.25–93.75]	77.34 ± 16.55	81.25 [64.06–93.75]	0.788
Emotional wellbeing	79.88 ± 17.30	81.25 [75.00–93.75]	79.10 ± 13.24	81.25 [68.75–87.50]	0.650
Self-esteem	57.03 ± 23.32	56.25 [40.63–73.44]	59.77 ± 17.31	62.50 [50.00–68.75]	0.616
Family	72.46 ± 17.09	71.88 [56.25–87.50]	66.02 ± 13.65	62.50 [56.25–75.00]	0.053
Social	73.05 ± 22.81	75.00 [56.25–92.18]	72.46 ± 20.44	75.00 [62.50–87.50]	0.772
School	63.09 ± 22.07	65.63 [51.56–79.69]	72.66 ± 16.01	75.00 [62.50–85.94]	0.022
Control group (n = 21)					
Kid-KINDL-R total score	69.69 ± 14.83	68.75 [62.50–79.69]	76.98 ± 9.84	77.08 [72.92–82.81]	0.008
Kid-KINDL-R subscales					
Physical wellbeing	80.95 ± 12.57	81.25 [75.00–87.50]	86.90 ± 13.10	87.50 [78.13–96.88]	0.047
Emotional wellbeing	77.38 ± 21.96	81.25 [68.75–93.75]	84.82 ± 13.34	87.50 [75.00–96.88]	0.210
Self-esteem	52.98 ± 26.85	43.75 [31.25–75.00]	64.29 ± 13.86	68.75 [53.13–75.00]	0.075
Family	68.45 ± 16.94	68.75 [56.25–87.50]	69.64 ± 14.29	82.50 [59.38–81.25]	0.627
Social	75.00 ± 16.54	75.00 [68.75–84.38]	78.27 ± 11.46	75.00 [75.00–84.38]	0.293
School	63.39 ± 18.15	56.25 [50.00–75.00]	77.98 ± 9.81	81.25 [75.00–84.38]	0.001

KINDL-R, Kinder Lebensqualität Fragebogen.

## Data Availability

The original contributions presented in the study are included in the article; further inquiries can be directed to the corresponding author.

## Supplementary Materials

The following supporting information can be downloaded at: https://www.mdpi.com/article/10.3390/jcm13226892/s1, Table S1: Comparison of Kid-KINDL-R scores between groups above and below the cut-off score for JSQ-ES scores in the epilepsy group.
