# Supplementary material for: Sleep Problems and Quality of Life in Children with Epilepsy Without Neurodevelopmental Disorders
Source: J Clin Med. 2024 Nov 16;13(22):6892. doi: 10.3390/jcm13226892 (PMC11594748; doi:10.3390/jcm13226892)
Supplement: Supplementary file 1 [file jcm-13-06892-s001.zip › jcm-3206656-supplementary.pdf]

Supplemental Table S1. Comparison of Kid-KINDL-R scores between groups above and below the cut-off score for JSQ-ES scores in the epilepsy group

	JSQ-ES									
	Total score		Restless legs syndrome		Sleep disordered breathing		Morning symptoms		Nighttime awakenings	
	<80 (*n*=26)<br>≥80 (*n*=6)	*p*	<8 (*n*=24)<br>≥8 (*n*=8)	*p*	<10 (*n*=20)<br>≥10 (*n*=12)	*p*	<9 (*n*=14)<br>≥9 (*n*=18)	*p*	<8 (*n*=19)<br>≥8 (*n*=13)	*p*
Child-self report										
Kid-KINDL-R total score	69.19 ± 14.75<br>73.44 ± 10.31	0.424	70.61 ± 3.10<br>68.10 ± 3.57	0.601	69.64 ± 14.10<br>70.57 ± 14.39	0.859	63.84 ± 16.54<br>74.77 ± 9.59	0.040*	68.64±14.57<br>71.96±13.40	0.513
Kid-KINDL-R subscales										
Physical wellbeing	74.52 ± 19.68<br>73.96 ± 22.85	0.957	75.52 ± 20.43<br>71.09 ± 19.17	0.588	75.63 ± 19.65<br>72.40 ± 21.06	0.671	66.96 ± 22.12<br>80.21 ± 16.36	0.061	76.64 ± 21.83<br>71.15 ± 17.03	0.432
Emotional Wellbeing	79.33 ± 17.39<br>82.29 ± 18.29	0.728	79.43 ± 18.10<br>81.25 ± 15.67	0.788	79.38 ± 13.92<br>80.73 ± 22.53	0.834	76.79 ± 20.28<br>82.29 ± 14.74	0.401	79.61 ± 16.90<br>80.29 ± 18.55	0.916
Self-esteem	55.53 ± 25.02<br>63.54 ± 13.36	0.293	57.55 ± 25.27<br>55.47 ± 17.50	0.799	56.56 ± 24.96<br>57.81 ± 21.34	0.882	50.00 ± 25.94<br>62.50 ± 20.11	0.150	51.64 ± 25.16<br>64.90 ± 18.49	0.096
Family	69.95 ± 16.87<br>83.33 ± 14.61	0.084	70.83 ± 17.06<br>77.34 ± 17.34	0.374	65.00 ± 15.63<br>84.90 ± 11.45	<0.001<br>***	69.64 ± 18.65<br>74.65 ± 15.97	0.430	70.07 ± 15.81<br>75.96 ± 18.90	0.365
Social	74.52 ± 22.43<br>66.67 ± 25.52	0.510	75.52 ± 23.31<br>65..63 ± 20.86	0.279	76.25 ± 20.84<br>67.71 ± 25.82	0.343	65.18 ± 25.44<br>79.17 ± 19.05	0.099	74.01 ± 23.78<br>71.63 ± 22.19	0.775
School	61.30 ± 23.45<br>70.83 ± 13.50	0.207	64.84 ± 23.30<br>57.81 ± 18.22	0.393	65.00 ± 23.24<br>59.90 ± 20.55	0.523	54.46 ± 27.46<br>69.79 ± 14.26	0.050	59.87 ± 23.51<br>67.79 ± 19.74	0.311
Parent proxy-report										
Kid-KINDL-R total score	72.12 ± 12.07<br>67.36 ± 10.51	0.359	71.13 ± 12.13<br>71.48 ± 11.48	0.943	72.81 ± 11.10<br>68.58 ± 12.91	0.355	74.18 ± 13.67<br>68.92 ± 9.88	0.216	72.86 ± 12.12<br>68.83 ± 11.33	0.345
Kid-KINDL-R subscales										
Physical wellbeing	76.92 ± 15.79<br>79.17 ± 21.16	0.815	76.56 ± 16.20<br>79.69 ± 18.53	0.679	78.44 ± 16.78<br>75.52 ± 16.74	0.638	79.46 ± 15.59<br>75.69 ± 17.53	0.532	80.26 ± 16.70<br>73.08 ± 16.01	0.231
Emotional Wellbeing	80.53 ± 13.27<br>72.92 ± 12.29	0.215	79.95 ± 13.67<br>76.56 ± 12.39	0.526	81.25 ± 13.14<br>75.52 ± 13.18	0.245	82.14 ± 13.17<br>76.74 ± 13.18	0.259	81.58 ± 14.05<br>75.48 ± 11.54	0.190
Self-esteem	60.10 ± 18.46<br>58.33 ± 12.29	0.781	58.07 ± 19.02<br>64.84 ± 9.99	0.209	62.50 ± 12.50<br>55.21 ± 23.21	0.255	64.73 ± 18.44<br>55.90 ± 15.82	0.156	59.54 ± 19.02<br>60.10 ± 15.21	0.928
Family	67.07 ± 14.64<br>61.46 ± 7.31	0.194	66.93 ± 14.68<br>63.28 ± 10.26	0.449	65.00 ± 12.40<br>67.71 ± 15.95	0.620	68.30 ± 11.87<br>64.24 ± 14.98	0.412	69.08 ± 13.58<br>61.54 ± 12.97	0.125
Social	74.28 ± 21.89<br>64.58 ± 10.21	0.123	72.66 ± 21.71<br>71.88 ± 17.36	0.919	74.69 ± 20.73<br>68.75 ± 20.30	0.435	72.32 ± 27.49<br>72.57 ± 13.58	0.976	74.67 ± 21.70<br>69.23 ± 18.83	0.457
School	73.80 ± 15.00<br>67.71 ± 20.70	0.520	72.66 ± 15.06<br>72.66 ± 19.75	1.000	75.00 ± 15.04<br>68.75 ± 17.48	0.293	78.13 ± 17.64<br>68.40 ± 13.64	0.089	72.04 ± 14.79<br>73.56 ± 18.25	0.806

	JSQ-ES									
	Insomnia		Excessive daytime sleepiness		Daytime behaviors		Sleep habits		Irregular/delayed sleep phase	
	<4 (*n*=25)<br>≥4 (*n*=7)	*p*	<8 (*n*=19)<br>≥8 (*n*=13)	*p*	<11 (*n*=15)<br>≥11 (*n*=17)	*p*	<7 (*n*=20)<br>≥7 (*n*=12)	*p*	<12 (*n*=21)<br>≥12 (*n*=11)	*p*
Child-self report										
Kid-KINDL-R total score	69.08 ± 15.27<br>73.21 ± 7.88	0.499	68.75 ± 14.85<br>71.79 ± 12.98	0.544	66.53 ± 14.19<br>73.04 ± 13.48	0.195	68.33 ± 14.85<br>72.74 ± 12.53	0.377	70.29 ± 14.03<br>69.41 ± 14.56	0.872
Kid-KINDL-R subscales										
Physical	75.75 ± 20.60	0.456	77.96 ± 20.13	0.226	72.92 ± 22.49	0.701	71.88 ± 18.64	0.384	75.30 ± 19.81	0.741

wellbeing	69.64 ± 17.83		69.23 ± 19.17		75.74 ± 17.94		78.65 ± 22.05		72.73 ± 20.97	
Emotional Wellbeing	80.25 ± 17.74<br>78.57 ± 16.87	0.823	80.59 ± 16.52<br>78.85 ± 19.02	0.790	79.17 ± 17.62<br>80.51 ± 17.52	0.830	76.25 ± 19.51<br>85.94 ± 11.03	0.083	82.44 ± 14.20<br>75.00 ± 22.01	0.326
Self-esteem	55.25 ± 24.59<br>63.39 ± 18.20	0.353	51.32 ± 25.22<br>65.38 ± 17.97	0.075	47.50 ± 22.51<br>65.44 ± 21.21	0.028*	56.25 ± 23.82<br>58.33 ± 23.44	0.811	55.95 ± 23.67<br>59.09 ± 23.61	0.725
Family	71.75 ± 17.78<br>75.00 ± 15.31	0.642	67.11 ± 17.16<br>80.29 ± 14.17	0.025*	70.00 ± 16.57<br>74.63 ± 17.74	0.451	74.69 ± 14.55<br>68.75 ± 20.81	0.350	69.35 ± 17.78<br>78.41 ± 14.62	0.136
Social	70.75 ± 24.39<br>81.25 ± 14.43	0.170	73.68 ± 23.53<br>72.12 ± 22.62	0.851	70.00 ± 24.80<br>75.74 ± 21.30	0.491	72.19 ± 23.95<br>74.48 ± 21.73	0.783	72.92 ± 22.74<br>73.30 ± 24.06	0.966
School	60.75 ± 24.24<br>71.43 ± 7.95	0.265	61.84 ± 22.91<br>64.90 ± 21.58	0.704	59.58 ± 23.37<br>66.18 ± 21.09	0.411	58.75 ± 21.69<br>70.31 ± 21.67	0.157	65.77 ± 21.53<br>57.95 ± 23.23	0.365
Parent proxy-report										
Kid-KINDL-R total score	72.67 ± 11.59<br>66.07 ± 11.86	0.222	72.09 ± 11.47<br>69.95 ± 12.59	0.629	73.61 ± 12.41<br>69.12 ± 11.15	0.293	70.10 ± 12.02<br>73.09 ± 11.66	0.495	72.07 ± 10.86<br>69.60 ± 13.80	0.613
Kid-KINDL-R subscales										
Physical wellbeing	80.50 ± 14.01<br>66.07 ± 21.00	0.127	80.26 ± 13.05<br>73.08 ± 20.47	0.234	81.25 ± 13.15<br>73.90 ± 18.78	0.206	75.31 ± 16.78<br>80.73 ± 16.31	0.377	79.46 ± 17.36<br>73.30 ± 14.81	0.303
Emotional Wellbeing	80.00 ± 12.50<br>75.89 ± 16.31	0.554	80.59 ± 12.65<br>76.92 ± 14.29	0.463	82.08 ± 15.10<br>76.47 ± 11.16	0.248	77.81 ± 12.25<br>81.25 ± 15.08	0.512	79.17 ± 13.01<br>78.98 ± 14.33	0.971
Self-esteem	60.50 ± 19.16<br>57.14 ± 8.41	0.507	57.57 ± 18.47<br>62.98 ± 15.62	0.379	57.08 ± 21.24<br>62.13 ± 13.16	0.434	56.56 ± 19.81<br>65.10 ± 10.81	0.126	59.52 ± 16.13<br>60.23 ± 20.21	0.922
Family	67.00 ± 13.80<br>62.50 ± 13.50	0.456	67.43 ± 14.67<br>63.94 ± 12.28	0.472	70.83 ± 15.79<br>61.76 ± 10.10	0.069	67.50 ± 14.57<br>63.54 ± 12.16	0.416	64.58 ± 14.30<br>68.75 ± 12.50	0.403
Social	72.75 ± 22.60<br>71.43 ± 10.74	0.830	73.36 ± 23.83<br>71.15 ± 15.00	0.751	76.25 ± 22.19<br>69.12 ± 18.81	0.339	72.81 ± 19.27<br>71.88 ± 23.16	0.907	74.70 ± 20.58<br>68.18 ± 20.44	0.402
School	75.25 ± 15.36<br>63.39 ± 15.91	0.112	73.36 ± 15.57<br>71.63 ± 17.23	0.776	74.17 ± 17.34<br>71.32 ± 15.16	0.627	70.63 ± 11.84<br>76.04 ± 21.46	0.363	75.00 ± 13.83<br>68.18 ± 19.46	0.317

* p<0.05; *** p<0.001; Unless indicated otherwise, data are given as the mean ± SD. JSQ-ES, Japanese Sleep Questionnaire for Elementary Schoolers; KINDL-R, Kinder Lebensqualität fragebogen.
